# An exploratory study of factors associated with long-term, high-dose opioid prescription in cancer patients in Japan based on a medical claims database

**DOI:** 10.1007/s00520-022-07121-3

**Published:** 2022-05-11

**Authors:** Tatsuya Hashimoto, Hirokazu Mishima, Chika Sakai, Yuichi Koretaka, Yoji Saito

**Affiliations:** 1 Department of Anesthesiology, Faculty of Medicine, Shimane University, 89-1 Enya-cho, Izumo, Shimane Japan; 2 Medical Affairs Department, Shionogi & Co. Ltd., Osaka, Japan; 3 Data Science Department, Shionogi & Co. Ltd., Osaka, Japan

**Keywords:** Cancer pain, Long-term high-dose opioid prescription, Cancer survivor, Claims database

## Abstract

**Purpose:**

As the cancer survivors increase, patients using long-term and high-dose opioids are also increasing. Therefore, the promotion of appropriate use is important. This study investigated the actual status of opioid prescriptions in Japan and identified factors associated with long-term, high-dose prescription.

**Methods:**

We conducted a case-control study using a hospital-based administrative claims database. Patients with a diagnosis of cancer and prescriptions of opioids were included. Patients who received continuous opioid for less than 183 days were defined as the “control,” and patients who received continuous opioid at higher dose levels (≥ 120 mg/day of oral morphine equivalent) for 183 days or more were defined as the “case.” The case was subdivided into two groups: those with the duration of less than 730 days (case I) and 730 days or more (case II). After describing factors possibly associated with long-term, high-dose opioid prescription, ordinal logistic regression analysis was conducted.

**Results:**

We included 19,176 patients; of these, 13,517 were in the control, 111 were in the case I, and 682 were in the case II. The analysis showed that distant metastasis, back pain, dose of opioids, non-opioid analgesics, prescription, and chemotherapy during the opioid prescriptions were significantly associated with long-term, high-dose opioid prescription.

**Conclusion:**

Four percent of the study population were prescribed long-term, high-dose opioids, and several comorbidities and concomitant medications were identified as associated factors. Opioids might be also prescribed for non-cancer chronic pain. It is necessary to properly distinguish the type of pain and to use opioids safely and appropriately.

**Supplementary Information:**

The online version contains supplementary material available at 10.1007/s00520-022-07121-3.

## Introduction

In Japan, the population of cancer patients is growing rapidly with the aging of the population, and the survival time of patients is becoming longer due to progress in early diagnosis and treatment [1, 2].

Cancer pain is associated with decreased quality of life and poor adherence to cancer treatment; therefore, appropriate pain control with opioids is important. When cancer patients survive for a long time, their cancer pain are often complicated by non-cancer pain, and long-term opioid use requires pain-specific treatment while also considering treatment of breakthrough pain [3].

On the other hand, long-term or high-dose prescriptions are more likely to cause the adverse effects such as opioid-induced hyperalgesia, gonadal dysfunction, intestinal dysfunction, cognitive dysfunction, sleep disorders, immune disorders, and addiction. Appropriate use of opioids is also important to prevent the occurrence of an opioid crisis [3].

The study by Jones et al. reviewed the literatures investigating the factors associated with long-term opioid therapy for cancer survivors, and describes a relationship between long-term opioid use and important biopsychosocial factors such as cancer sites, socioeconomic factors, and comorbidities [4]. Whereas although Azuma et al. reported a nationwide survey of opioid use in Japan [5], there are few reports on the actual status of opioid prescriptions among cancer patients in Japan, and the factors that lead to long-term, high-dose prescriptions are not clear. Azuma et al. reported that the adequacy of opioid analgesic consumption was lower in Japan than in the USA or European countries; however, the survival time of cancer survivors is increasing and there is concern that long-term, high-dose prescriptions of opioids might become a problem in Japan. Therefore, it is essential to identify the factors associated with long-term, high-dose opioid use and to lead to appropriate use.

In the present study, we investigated the situation of cancer patients in Japan prescribed opioids and the factors associated with long-term, high-dose prescriptions. We also investigated exploratory associations between rescue medications, which are often used for breakthrough pain, and long-term, high-dose prescriptions of opioids.

## Methods

### Study design and data source

This study was designed as a case-control study using a hospital-based administrative claims database in Japan constructed by Medical Data Vision Co., Ltd (MDV) [6]. The MDV claims database contains over 30 million patient demographics (age, sex), ICD-10 coded diagnoses, medical practices, hospitalizations, prescribed medications, and drug prices from over 400 hospitals with an acute inpatient care system called Diagnostic Procedure Combination (DPC) covering 22% of Japanese hospitals with a DPC system.

### Study population

From the MDV claims database, we identified the eligible patients by the following three criteria: (i) having diagnosis of cancer between April 2008 and July 2020 (ICD-10 codes shown in the Supplement), (ii) having at least one prescription of any of the following opioids: morphine, fentanyl, oxycodone, tapentadol, hydromorphine, methadone, and/or buprenorphine on or after the date of the first diagnosis of cancer; and (iii) could be followed for at least 730 days. The definition of opioids was based on the Clinical Guidelines for Cancer Pain Management [7]. The injectable preparations were excluded from the definition of opioid because they are not expected to be utilized for the long-term pain control, and buprenorphine transdermal patches (Norspan Tape®) are not indicated for cancer pain in Japan. To enroll patients for whom the duration of opioid prescription could be assessed, the eligibility criteria for the follow-up period were considered. The follow-up period was defined as the duration between the index date which was the date of the first prescription of opioids after the initial diagnosis of cancer and the date when the patient died or the last record on his/her care was available.

### Analysis population

The analysis population was defined based on the duration of opioid prescription and the mean opioid dose during the prescription period. The definitions of the cut-off values for long term and high dose were based on previous studies. The definition of long-term opioid prescription was defined by referring to the clinical guideline for non-cancer pain [8]. The guideline states that “after 6 months of opioid treatment with a good response, a dose reduction or drug holiday should be discussed with the patient.” Since the target patients of this study were cancer patients but the pain was not limited to cancer pain, patients who were prescribed the drug for more than 6 months (183 days) were defined as long term. The definition of high-dose opioid use follows that of “Guidance on the appropriate use of medical narcotics” published by Ministry of Health, Labour, and Welfare of Japan and “Guidelines for Pharmacologic Management of Neuropathic Pain” by Japan Society of Pain Clinicians [3, 9], which define ≥120 mg per day of oral morphine equivalence as a high dose.

Patients for whom opioids were prescribed continuously for less than 183 days were allocated to the control group, and patients having received prescription of opioids for 183 days or more at the mean dose level ≥120 mg per day of oral morphine equivalence were allocated to the case group. To assess the impact of further long-term prescriptions, the case group was subdivided into two groups according to the opioid prescription period: those with the prescription period of less than 730 days (case I) and 730 days or more (case II).

**Fig. 1:**
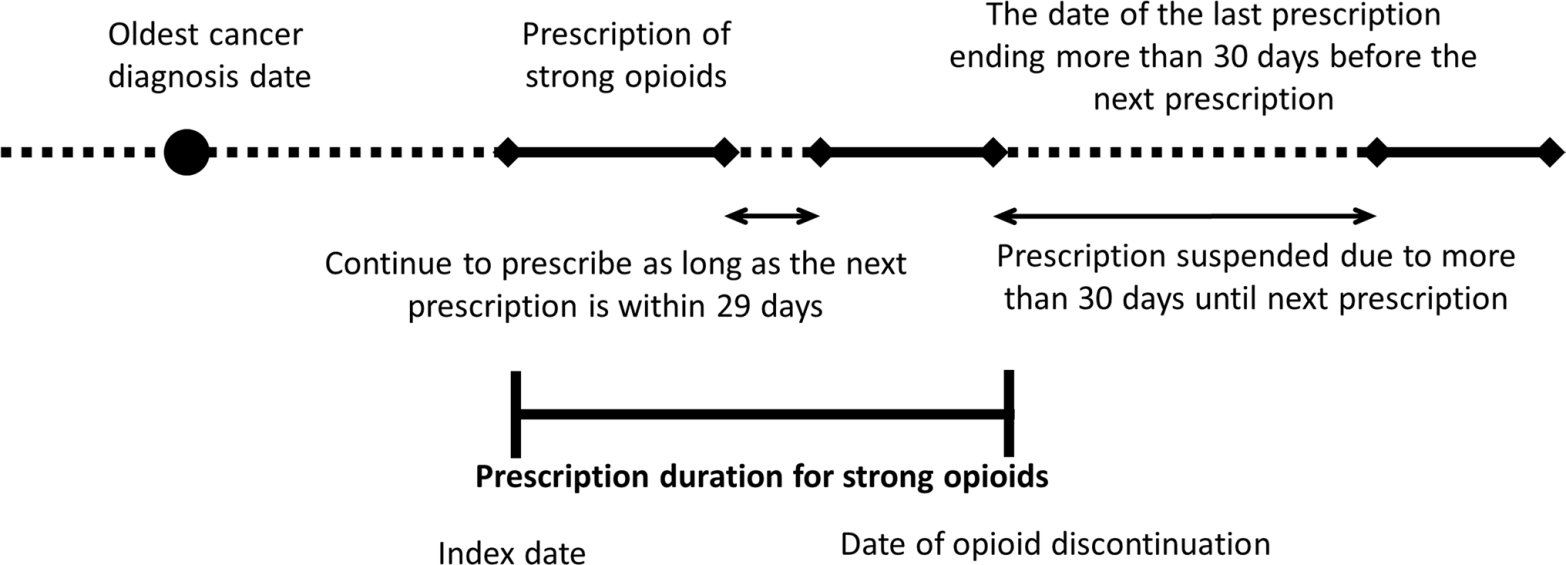
Definition of duration of opioid prescription

The prescription period was defined as the duration from the index date to the date of death or end of the prescription. The end of prescription was defined as an interval of 30 days or more between prescriptions of the relevant opioid after the index date, and the last day covered by the last prescription was defined as the day of end of opioid prescription. Even when the opioid was changed to other opioid(s) or combined with additional opioid(s), the prescription was considered to be continued (Fig. 1).

The mean opioid dose during the prescription period was calculated by dividing the active ingredient’s quantity equivalent in potency to the oral-dose morphine hydrochloride hydrate 30 mg (an opioid) prescribed during the prescription period [3, 7] by the prescription period.

### Statistical analysis

#### Factors associated with long-term, high-dose prescription of opioid

The following factors possibly associated with long-term, high dose prescription of opioids were described separately for the case groups and the control group: gender, age, site of cancer, metastasis, comorbidities, dose of opioids, duration from diagnosis of cancer to the start of opioid prescription, prescription of pain-related drugs other than opioids, and types of cancer treatment. Prescription of pain-related medication other than opioids and types of cancer treatment were evaluated both before and after the start of opioid prescription. Each case group was compared with the control group in a multiple comparison using the Dunnett test for continuous variables and the Steel test for categorical variables.

On the basis of the descriptive statistics, ordinal logistic regression analysis was conducted to investigate the factors associated with long-term, high-dose prescription of opioids. The control group, case I group, and case II group were considered as dependent variables. Through this analysis, the odds ratio of long-term, high-dose prescription of opioids for each explanatory variable and its 95% confidence interval were calculated. The explanatory variables were selected by the following steps. Step 1: selection of variables of descriptive statistics showing significant differences both between the control group and case I group and between the control group and case II group (*p*<0.05) as well as categorical variables whose incidence in the case or control group was 5% or higher. Step 2: variables selected from step 1 were re-evaluated for co-linearity and clinical validity.

#### Assessment of the rescue medication prescription

To investigate the actual status of rescue medication, the present study additionally evaluated the rescue medication during the opioid prescription in each of the case and control groups. The rescue medication prescription was defined as prescription of oxycodone (Oxinorm®) powder, oral immediate release tablet, hydromorphone (Narurapid®) tablets, fentanyl (Abstral®) sublingual tablets, fentanyl (E-fen®) buccal tablets, morphine (Anpec®) suppositories, morphine (Opso®) oral solution, or morphine hydrochloride immediate release tablet. The percentage of patients who received rescue medication during the prescription period of opioid was calculated. In addition, among patients who received rescue medication, the mean daily number of rescue medication use, which is the total number of rescue medication use during the opioid prescription period divided by the prescription period, and the mean rescue medication dose, which is the total doses of rescue medication during the opioid prescription period divided by the opioid prescription period, were described. Each case group was compared with the control group in a multiple comparison using the Steel test for percentage of patients receiving rescue medication and the Dunnett test for the mean daily number of rescue medication use and the mean rescue medication dose.

For all calculations of oral morphine equivalent dose, fentanyl sublingual tablets and fentanyl buccal tablets were excluded from the calculation because they are not defined in the guidelines [3, 7] and cannot be converted oral morphine equivalent dose.

All data analyses were carried out with SAS v.9.4, and *P* values of <0.05 were considered significant.

## Results

### Analysis population

**Fig. 2:**
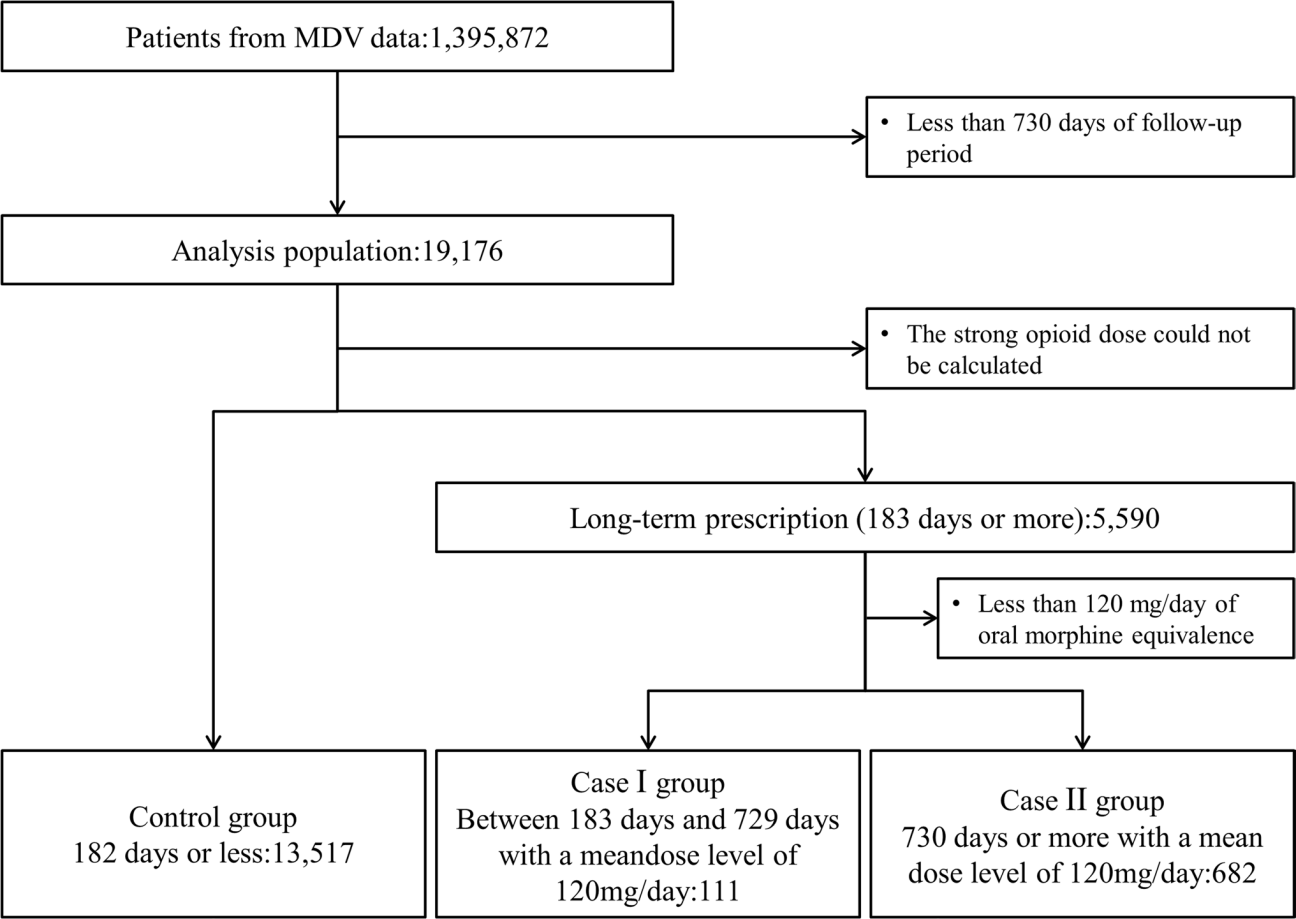
Patient flow chart

The patient flow chart is shown in Fig. 2. From the database, 19,176 patients were eligible for the study, and of these, the opioid dose could be calculated in 19,107. There were 13,517 (70.7%) in the control, 111 (0.6%) in the case I, and 682 (3.5%) in the case II group. Supplement shows the distribution of opioid dose by group.

### Factors associated with long-term, high-dose prescription of opioid

Table 1 shows the results of descriptive analysis of all factors examined as factors relevant to long-term, high-dose prescription, with detailed definitions of each factor provided in the Supplement. In the evaluation of patient background and cancer site and metastasis and comorbidities, there were significant differences in age, esophageal cancer, breast cancer, bone metastasis, distant metastasis, back pain, schizophrenia, emotional disorder, and neurological disorder in both comparisons between the control and case I groups and between the control and case II groups. In the evaluation of the period prior to start opioid prescription, duration from cancer diagnosis to start of opioid prescription, prescription of non-opioid analgesics, chemotherapy, and radiotherapy were significantly different in both comparisons. In the evaluation during opioid prescription, mean opioid dose and prescription of non-opioid analgesics, tricyclic antidepressant (TCA), selective serotonin reuptake inhibitor (SSRI), serotonin noradrenaline reuptake inhibitor (SNRI), gabapentinoids, anticonvulsants, and central muscle relaxants were significantly different in both comparisons.

**Table 1:** Evaluation of factors associated with long-term prescription

	Control *n*=13,517	Case I *n*=111		Case II *n*=682	
	*n* (%)	*n* (%)	*p*	*n* (%)	*p*
Male	8460 (62.6)	61 (55.0)	0.186	342 (50.1)	<0.0001
Age (SD)	64.5 (12.6)	60.5 (11.9)	0.002	60.9 (11.1)	<0.0001
Cancer site					
Lip, oral cavity, and pharynx	2320 (17.2)	11 (9.9)	0.085	15 (2.2)	<0.0001
Esophagus	749 (5.5)	0 (0.0)	0.021	5 (0.7)	<0.0001
Stomach	722 (5.3)	2 (1.8)	0.186	24 (3.5)	0.073
Small intestine	28 (0.2)	3 (2.7)	<.0001	0 (0.0)	0.413
Large intestine, anus, and anal	1355 (10.0)	13 (11.7)	0.803	127 (18.6)	<0.0001
Liver and intrahepatic bile ducts	320 (2.4)	1 (0.9)	0.524	10 (1.5)	0.239
Gallbladder and unspecified parts of biliary tract	147 (1.1)	0 (0.0)	0.466	10 (1.5)	0.585
Pancreas	315 (2.3)	2 (1.8)	0.918	39 (5.7)	<0.0001
Other and ill-defined digestive organs	5 (0.0)	0 (0.0)	0.974	0 (0.0)	0.852
Respiratory and intrathoracic organs	2568 (19.0)	21 (18.9)	1.000	97 (14.2)	0.004
Bone and articular cartilage	91 (0.7)	0 (0.0)	0.623	11 (1.6)	0.009
Melanoma and other malignant neoplasms of skin	59 (0.4)	0 (0.0)	0.735	1 (0.1)	0.445
Mesothelial and soft tissue	143 (1.1)	3 (2.7)	0.179	16 (2.3)	0.004
Breast	1031 (7.6)	19 (17.1)	0.0004	117 (17.2)	<0.0001
Female genital organs	537 (4.0)	6 (5.4)	0.689	23 (3.4)	0.677
Male genital organs	608 (4.5)	3 (2.7)	0.594	44 (6.5)	0.034
Urinary tract	533 (3.9)	10 (9.0)	0.013	36 (5.3)	0.159
Eye, brain, and other parts of central nervous system	18 (0.1)	1 (0.9)	0.061	0 (0.0)	0.565
Thyroid and other endocrine glands	133 (1.0)	1 (0.9)	0.995	5 (0.7)	0.765
Ill-defined, other secondary, and unspecified sites	2841 (21.0)	29 (26.1)	0.342	195 (28.6)	<0.0001
Lymphoid, hematopoietic, and related tissue	1758 (13.0)	15 (13.5)	0.984	84 (12.3)	0.841
Independent (primary) multiple sites	0 (0.0)	0 (0.0)	1.000	0 (0.0)	1.000
Oral cavity, esophagus, and stomach	0 (0.0)	0 (0.0)	1.000	0 (0.0)	1.000
Other and unspecified digestive organs	0 (0.0)	0 (0.0)	1.000	0 (0.0)	1.000
Middle ear and respiratory system	0 (0.0)	0 (0.0)	1.000	0 (0.0)	1.000
Melanoma	1 (0.0)	0 (0.0)	0.995	0 (0.0)	0.968
Skin	4 (0.0)	1 (0.9)	<0.0001	0 (0.0)	0.880
Breast	0 (0.0)	0 (0.0)	1.000	0 (0.0)	1.000
Cervix uteri	13 (0.1)	0 (0.0)	0.934	0 (0.0)	0.661
Other and unspecified genital organs	1 (0.0)	0 (0.0)	0.995	0 (0.0)	0.968
Other and unspecified sites	5 (0.0)	0 (0.0)	0.974	0 (0.0)	0.852
Metastatic cancer					
Bone metastasis	2054 (15.2)	48 (43.2)	<0.0001	278 (40.8)	<0.0001
Secondary malignant neoplasm	4332 (32.0)	69 (62.2)	<0.0001	431 (63.2)	<0.0001
Comorbidities					
Herpes zoster	962 (7.1)	11 (9.9)	0.445	57 (8.4)	0.393
Osteoarthritis of knee	449 (3.3)	4 (3.6)	0.983	42 (6.2)	0.0002
Back pain	5964 (44.1)	75 (67.6)	<0.0001	441 (64.7)	<0.0001
Spinal stenosis	669 (4.9)	7 (6.3)	0.762	59 (8.7)	<0.0001
Spondylosis	1093 (8.1)	7 (6.3)	0.743	89 (13.0)	<0.0001
Other disorders of bone	33 (0.2)	0 (0.0)	0.842	2 (0.3)	0.960
Alcohol dependence	39 (0.3)	0 (0.0)	0.816	0 (0.0)	0.295
Other psychoactive substance dependence	2 (0.0)	0 (0.0)	0.990	0 (0.0)	0.938
Schizophrenia	1519 (11.2)	29 (26.1)	<0.0001	144 (21.1)	<0.0001
Mood disorders	1642 (12.1)	32 (28.8)	<0.0001	198 (29.0)	<0.0001
Anxiety	2331 (17.2)	36 (32.4)	<0.0001	181 (26.5)	<0.0001
Sleep disorders	29 (0.2)	0 (0.0)	0.859	4 (0.6)	0.096
Delirium due to known physiological condition	292 (2.2)	4 (3.6)	0.508	8 (1.2)	0.154
Dementia	77 (0.6)	0 (0.0)	0.670	5 (0.7)	0.826
Diabetes mellitus	3969 (29.4)	36 (32.4)	0.729	187 (27.4)	0.476
Hepatic disorder	2006 (14.8)	16 (14.4)	0.990	82 (12.0)	0.084
Chronic kidney disease	2067 (15.3)	15 (13.5)	0.843	86 (12.6)	0.110
Patterns of opioid prescription					
Mean prescription dose per day (SD)	26.6 (82.3)	219.6 (169.9)	<0.0001	247.5 (180.7)	<0.0001
Mean number of prescriptions per day (SD)	533.0 (1099.1)	860.8 (1260.1)	0.004	930.2 (1249.8)	<0.0001
Use of analgesics other than opioids (before start opioids)					
Non-opioid analgesics	10,256 (75.9)	49 (44.1)	<0.0001	339 (49.7)	<0.0001
TCA	77 (0.6)	1 (0.9)	0.874	8 (1.2)	0.090
SSRI	99 (0.7)	2 (1.8)	0.345	10 (1.5)	0.063
SNRI	136 (1.0)	1 (0.9)	0.992	8 (1.2)	0.892
Gabapentinoid	1008 (7.5)	9 (8.1)	0.958	81 (11.9)	<0.0001
Antiepileptic	314 (2.3)	5 (4.5)	0.243	19 (2.8)	0.682
Antiarrhythmic	516 (3.8)	3 (2.7)	0.789	9 (1.3)	0.001
NMDA receptor antagonist	623 (4.6)	1 (0.9)	0.121	19 (2.8)	0.050
Centrally acting muscle relaxant	369 (2.7)	3 (2.7)	1.000	18 (2.6)	0.987
Use of analgesics other than opioids (during opioid prescription)					
Non-opioid analgesics	10,696 (79.1)	106 (95.5)	<0.0001	670 (98.2)	<0.0001
TCA	131 (1.0)	5 (4.5)	0.0004	62 (9.1)	<0.0001
SSRI	131 (1.0)	6 (5.4)	<0.0001	44 (6.5)	<0.0001
SNRI	203 (1.5)	22 (19.8)	<0.0001	166 (24.3)	<0.0001
Gabapentinoid	1395 (10.3)	52 (46.8)	<0.0001	455 (66.7)	<0.0001
Antiepileptic	457 (3.4)	22 (19.8)	<0.0001	181 (26.5)	<0.0001
Antiarrhythmic	336 (2.5)	9 (8.1)	0.0003	70 (10.3)	<0.0001
NMDA receptor antagonist	353 (2.6)	6 (5.4)	0.130	81 (11.9)	<0.0001
Centrally acting muscle relaxant	333 (2.5)	11 (9.9)	<0.0001	53 (7.8)	<0.0001
Cancer treatment (before start opioids)					
Operative treatment	684 (5.1)	2 (1.8)	0.222	11 (1.6)	<0.0001
Radiotherapy	4348 (32.2)	16 (14.4)	0.0001	49 (7.2)	<0.0001
Chemotherapy	6935 (51.3)	36 (32.4)	0.0001	230 (33.7)	<0.0001
Nerve block	278 (2.1)	2 (1.8)	0.978	12 (1.8)	0.834
Cancer treatment (during opioid prescription)					
Operative treatment	417 (3.1)	1 (0.9)	0.334	10 (1.5)	0.031
Radiotherapy	5171 (38.3)	41 (36.9)	0.950	161 (23.6)	<0.0001
Chemotherapy	9310 (68.9)	85 (76.6)	0.155	556 (81.5)	<0.0001
Nerve block	271 (2.0)	5 (4.5)	0.121	27 (4.0)	0.001

*SD*, standard deviation; *TCA*, tricyclic antidepressants; *SSRI*, selective serotonin reuptake inhibitor; *SNRI*, serotonin noradrenaline reuptake inhibitor; *NMDA*, N-methyl-D-aspartate

Table 2 shows the explanatory variables included in the ordinal logistic regression analysis and the results of analysis. Among the factors that showed significant differences in both comparisons in the descriptive statistics, bone metastasis was excluded from the ordinal logistic analysis because it was considered to be related to distant metastasis, and schizophrenia, emotional disorder, and neurological disorder were also excluded because they were considered to be related to TCA, SSRI, and SNRI. In addition, of the factors that did not meet the criteria in step 1 for selecting variables, cancer site of large intestine, anus, and anal, osteoarthritis of knee, spinal stenosis, spondylosis, radiotherapy, and chemotherapy during opioid prescription were included as explanatory variables from a clinical perspective. In the patient background, back pain (OR 1.346, 95% CI 1.068–1.697) and distant metastasis (OR 1.802, 1.422–2.285) showed a significant association with long-term, high-dose opioid prescription. The variables evaluated during opioid prescription include mean dose of opioids (OR 1.022, 1.021–1.024), non-opioid analgesics (OR 8.157, 4.207–15.815), SSRI (OR 1.990, 1.045–3.472), SNRI (OR 2.432, 1.704–3.472), gabapentinoids (OR 4.595, 3.648–5.788), anticonvulsants (OR 2.804, 2.049–3.837), and chemotherapy (OR 2.050, 1.509–2.785) showed a significant association with long-term, high-dose opioid prescription.

**Table 2:** Results of ordinal logistic regression analysis

Variable		Estimate	OR	95% CI		*p*
Intercept 1		−8.136	-	-	-	<0.0001
Intercept 2		−7.669	-	-	-	<.0001
Age		0.004	1.004	0.994	1.013	0.458
Cancer	Esophagus	−1.119	0.327	0.114	0.933	0.037
	Large intestine, anus, and anal	0.198	1.219	0.879	1.690	0.235
	Breast	0.345	1.412	0.994	2.004	0.054
Secondary malignant neoplasm		0.589	1.802	1.422	2.285	<0.0001
Concomitant disease	Osteoarthritis of knee	0.143	1.153	0.688	1.934	0.589
	Back pain	0.297	1.346	1.068	1.697	0.012
	Spinal stenosis	−0.009	0.991	0.656	1.497	0.964
	Spondylosis	0.060	1.062	0.731	1.543	0.752
Mean number of prescriptions per		0.022	1.022	1.021	1.024	<0.0001
Mean prescription dose per day		0.0001	1.000	1.000	1.000	0.034
Use of analgesics other than opioids (before start opioids)	Non-opioid analgesics	−0.789	0.454	0.349	0.591	<0.0001
Use of analgesics other than opioids (during opioid use)	Non-opioid analgesics	2.099	8.157	4.207	15.815	<0.0001
	TCA	0.459	1.583	0.906	2.765	0.107
	SSRI	0.688	1.990	1.045	3.789	0.036
	SNRI	0.889	2.432	1.704	3.472	<0.0001
	Gabapentinoid	1.525	4.595	3.648	5.788	<0.0001
	Antiepileptic	1.031	2.804	2.049	3.837	<0.0001
	Antiarrhythmic	0.259	1.296	0.795	2.112	0.298
	Centrally acting muscle relaxant	0.063	1.065	0.633	1.793	0.813
Cancer treatment (before start opioids)	Radiotherapy	−0.502	0.605	0.407	0.900	0.013
	Chemotherapy	−0.181	0.835	0.626	1.113	0.218
Cancer treatment (during opioid use)	Radiotherapy	−0.458	0.632	0.479	0.834	0.001
	Chemotherapy	0.718	2.050	1.509	2.785	<0.0001

*TCA*, tricyclic antidepressants; *SSRI*, selective serotonin reuptake inhibitor; *SNRI*, serotonin noradrenaline reuptake inhibitor

### Assessment of the rescue medication prescription

Table 3 shows the status of rescue drug prescription in each group. The percentage of patients receiving rescue medication was significantly higher in both case I group and case II group than the control group. In the analysis of patients receiving rescue medication, the mean number of rescue drug doses per day was significantly higher in case I group (1.25 times) and case II group (1.51 times) than in the control group (1.04 times). The mean rescue drug dose per day was significantly higher in case I group (32.23 mg) and case II group (43.62 mg) than in the control group (8.24 mg).

**Table 3:** Prescription of rescue medication

	Control *n*=13,517	Case I *n*=111		Case II *n*=682	
	*n* (%)	*n* (%)	*p*	*n* (%)	*p*
Prescription status of rescue medication					
Rescue only	3183 (23.5)	0 (0.0)	<0.0001	4 (0.6)	<0.0001
Base opioid only	3499 (25.9)	5 (4.5)		18 (2.6)	
Base and rescue	6835 (50.6)	106 (95.5)		660 (96.8)	
Prescription frequency and dosage of rescue medication					
Mean number of prescriptions per day (SD)	1.04 (0.76)	1.25 (1.13)	0.034	1.51 (1.55)	<0.0001
Mean prescription dose per day (SD)	8.24 (16.42)	32.23 (36.51)	<0.0001	43.62 (67.87)	<0.0001

*SD*, standard deviation

## Discussion

The present study attempted to identify factors associated with long-term prescription of opioids at high-dose levels for cancer patients for the first time in Japan. As a result, there were 13,517 patients in the control group who were prescribed opioids for less than 183 days, while 111 and 682 patients in the case I and case II groups who were prescribed opioids for more than 183 days and averaged more than 120 mg/day, respectively. The majority of the study population fell into the control group, of which 70.1% averaged less than 30 mg/day, while about 4% of the study population was identified as being on long-term, high-dose prescriptions. In addition, distant metastasis, prescription of non-opioid analgesics, SSRI, SNRI, gabapentinoids, and anticonvulsants during the opioid prescription period, chemotherapy, and back pain were shown to be associated with long-term, high-dose opioid prescribing.

In the present study, although esophageal cancer, breast cancer, and colorectal cancer were considered as candidate factors associated with long-term, high-dose prescription of opioids based on descriptive statistics results, no significant association was found in the ordinal logistic regression analysis. Although cancer prognosis varies by race and environmental factors and is not generally comparable, several studies described the relationship between cancer type and long-term opioid prescription. A study by Jones et al. reviewing studies of long-term prescription of opioid in cancer survivors noted a high rate of long-term opioid therapy in head and neck cancer [4]. The head and neck cancer was not identified as an associated factor in the present study; Jones et al. included all cancer survivors, whereas the present study included only patients who were prescribed opioid, which might bring different results due to population differences from the present study. The study by Salz et al. found that chronic use of opioids among colorectal and lung cancer survivors exceeded chronic use among controls [10]. In the present study, the point estimate for colorectal cancer also showed an association with long-term use. However, there are limitations in comparing the results of the present study with those of Salz et al. because they are comparisons with controls in non-cancer patients. Since there are limited reports on cancer types, further studies are needed.

Patients with distant metastasis are more likely to have severe cancer, which is consistent with the report on opioid use in colorectal cancer patients by Chen et al. [11] that the more severe the stage, the longer the duration of opioid prescription.

Regarding the prescription of non-opioid analgesics, Murphy et al. reported an association between polypharmacy and long-term prescription of opioids [12], and it is possible that pain management using a combination of analgesics is being implemented in clinical practice. In addition, Desai et al. reported that mental health comorbidity increased the risk of opioid prescription in older breast cancer patients [13], and the association with tranquilizers has also been reported in a study of non-cancer patients by Hauser et al. [14]. Shah et al. also reported that patients with chemotherapy-induced peripheral neuropathy were associated with long-term opioid prescription [15]. The results of the present study are consistent with previous reports that SSRI and SNRI as antidepressants, gabapentinoids, and antiepileptic drugs that are sometimes used to treat neuropathic pain, and chemotherapy were shown to be factors associated with long-term, high-dose of opioids.

We were the first to examine back pain as a factor in long-term, high-dose opioid prescriptions and showed a significant association. The reasons that back pain was identified as a factor are considered to be as follows. Firstly, since patients with bone metastasis were also included in this study, it is possible that pain associated with bone metastasis was diagnosed as back pain. The percentage of patients with bone metastasis among those diagnosed with back pain was higher in case I (44.0%) and case II (42.6%) groups than in the control group (20.8%). Secondly, opioids might have been prescribed for non-cancer pain. When we confirmed the details of the diagnostic names related to back pain, the 90% patients in both the case and control groups were diagnosed with lower back pain (Supplement). Nakamura et al. also reported that about 15.4% of Japanese people had chronic musculoskeletal pain, of which 65% had lower back pain, a high percentage [16]. As advances in cancer treatment have improved survival rates for many types of cancer, the patients complaining of non-cancer chronic pain are likely to increase. In Japan, the use of opioids for non-cancer chronic pain is likely to be prolonged, and problems such as side effects of opioids could occur due to excessive pursuit of pain relief; therefore, appropriate use is important. However, opioids might actually be prescribed without sufficiently distinguishing between these types of pain [3].

We further exploratory evaluated the status of rescue medications. The percentage of patients receiving both periodical medication and rescue medication was higher in the case groups than in the control group, and the rescue drug dosing frequency and level per day were also higher in the case groups. Patients with long-term, high-dose prescription of opioid were shown to have a higher frequency of use and prescription of rescue medications; however, confounding factors could not be ruled out because patients background were not matched; thus, the result needs careful interpretation.

The present study using a large-scale, hospital-based administrative claims database involves limitations arising from database characteristics. First, the database employed for this study covered only the data at hospitals having introduced an acute inpatient care system. Because this database does not track data when patients visit to other hospitals, if opioids were prescribed at other hospitals, the dose and frequency of opioid administration may have been underestimated. Second, the opioid dose might be underestimated because fentanyl sublingual tablets and fentanyl buccal tablets are not reflected in the prescription dose. However, since most drugs can be converted to morphine, we believe that this issue will have a small impact on the results. Third, because the data from this database are based on payment-related information, it is not possible to capture whether the patient actually took the drug. Fourth, since we were unable to assess the severity and type of pain, we cannot discuss the clinical appropriateness of the opioid prescription.

## Conclusions

We investigated the situation of cancer patients prescribed opioids and the factors leading to long-term, high-dose prescription. Within the scope of the study using the claims data, it was found that about 4% of cancer patients prescribed opioids in Japan were prescribed long term and high doses. Some of the factors identified were similar to those previously reported; however, the study also found new risks, such as back pain. This suggests the potential existence of a situation in Japan where opioids are used in cancer patients with insufficient distinction between cancer pain and non-cancer chronic pain. In order to avoid the careless use of opioids, it is essential to be careful about comorbidities in cancer patients, to evaluate the pain in individual patients for distinction between these types of pain and to select analgesics including opioids tailored to individual patients.

## Supplementary Information

ESM 1 (PDF 189 KB)

## Data Availability

The data that support the findings of this study are available from Medical Data Vision Co., Ltd, but restrictions apply to the availability of these data, which were used under license for the current study, and so are not publicly available. Data are however available from the authors upon reasonable request and with permission of Medical Data Vision Co., Ltd.

## Code availability

Code sharing not applicable to this article as some or all code used during the study were provided by a third party.
