# Supplementary material for: An exploratory study of factors associated with long-term, high-dose opioid prescription in cancer patients in Japan based on a medical claims database
Source: Support Care Cancer. 2022 May 11;30(8):6879–88. doi: 10.1007/s00520-022-07121-3 (PMC9213265; doi:10.1007/s00520-022-07121-3)
Supplement: Supplementary file 1 — (PDF 189 KB) [file 520_2022_7121_MOESM1_ESM.pdf]

Supplementary Information

Article title: An Exploratory Study of Factors Associated with Long-Term, High-Dose Opioid Prescription in Cancer Patients in Japan Based on a Medical Claims Database

Journal name: *Supportive Care in Cancer*

Author names: Tatsuya Hashimoto, Hirokazu Mishima, Chika Sakai, Yuichi Koretaka, Yoji Saito

Correspondence to:

Tatsuya Hashimoto

Affiliation: Department of Anesthesiology, Shimane University Faculty of Medicine, 89-1 Enya-cho, Izumo, Shimane, Japan

Email: hashit@med.shimane-u.ac.jp

Supplement 1. ICD-10 codes for cancers included in the analysis

ICD 10 code	Terms
C00-C14	lip, oral cavity, and pharynx
C15-C26	esophagus
C30-C39	stomach
C40-C41	small intestine
C43-C44	large intestine, anus, and anal
C45-C49	liver and intrahepatic bile ducts
C50	gallbladder and unspecified parts of biliary tract
C51-C58	pancreas
C60-C63	other and ill-defined digestive organs
C64-C68	respiratory and intrathoracic organs
C69-C72	bone and articular cartilage
C73-C75	melanoma and other malignant neoplasms of skin
C76-C80	mesothelial and soft tissue
C81-C96	breast
C97	female genital organs
D00	male genital organs
D01	urinary tract
D02	eye, brain, and other parts of central nervous system
D03	thyroid and other endocrine glands
D04	ill-defined, other secondary, and unspecified sites
D05	lymphoid, hematopoietic, and related tissue
D06	independent (primary) multiple sites
D07	oral cavity, esophagus, and stomach
D09	other and unspecified digestive organs

Supplement 2. Distribution of mean prescription dose of opioids during the prescription period

	Control<br>n=13,517	Case I<br>n=111	Case II<br>n=682
<30 mg/day	9,480(70.1%)	-[a]	-[a]
30 - <60 mg/day	2,918(21.6%)	-[a]	-[a]
60 - <90 mg/day	748(5.5%)	-[a]	-[a]
90 - <120 mg/day	228(1.7%)	-[a]	-[a]
120 - <200 mg/day	114(0.8%)	79(71.2%)	366(53.7%)
200 - <300 mg/day	9(0.1%)	14(12.6%)	183(26.8%)
300 - <400 mg/day	8(0.1%)	9(8.1%)	61(8.9%)
400 - <500 mg/day	3(0.0%)	1(0.9%)	25(3.7%)
≥500 mg/day	9(0.1%)	8(7.2%)	47(6.9%)

[a] Case group is defined by ≥120 mg/day of oral morphine equivalence

Supplement 3. Definition of comorbidities

Comorbidities	Definition
herpes zoster	ICD 10 code: B02, G53
osteoarthritis of knee	ICD 10 code: M17
back pain	ICD 10 code: M54
spinal stenosis	ICD 10 code: M480
spondylosis	ICD 10 code: M47
other disorders of bone	ICD 10 code: M89
alcohol dependence	ICD 10 code: F102
other psychoactive substance dependence	ICD 10 code: F192
schizophrenia	ICD 10 code: F20-F29
mood disorders	ICD 10 code: F30-F39
anxiety	ICD 10 code: F40-F48
sleep disorders	ICD 10 code: F51
delirium due to known physiological condition	ICD 10 code: F05
dementia	ICD 10 code: F01-F03
diabetes mellitus	ICD 10 code: E10-E14
hepatic disorder	ICD 10 code: K769 or patients with AST or ALT of 100 or more in the period from the date of first diagnosis of cancer to 365 days after the index date
chronic kidney disease	ICD 10 code: M18 or patients with eGFR of less than 60 in the period from the date of first diagnosis of cancer to 365 days after the index date

Supplement 4. Definition of analgesics other than opioids

Analgesics other than opioids	ATC code	Generic name
Non-opioid analgesics	B01C1	Aspirin
	M01A1	Ibuprofen
	M01A1	Indomethacin
	M01A1	Etodolac
	M01A1	Diclofenac sodium
	M01A1	Naproxen
	M01A1	Flurbiprofen
	M01A1	Meloxicam
	M01A1	Loxoprofen sodium hydrate
	M01A3	Celecoxib
	N02B0	Aspirin
	N02B0	Acetaminophen
TCA	N06A9	Amitriptyline hydrochloride
	N06A9	Amoxapine
	N06A9	Imipramine hydrochloride
	N06A9	Clomipramine hydrochloride
	N06A9	Trimipramine maleate
	N06A9	Dosulepin hydrochloride
	N06A9	Nortriptyline hydrochloride
	N06A9	Lofepramine hydrochloride
SSRI	N06A4	Sertraline hydrochloride
	N06A4	escitalopram oxalate
	N06A4	Paroxetine hydrochloride hydrate
	N06A4	Fluvoxamine maleate
SNRI	N06A5	Duloxetine hydrochloride
	N06A5	Venlafaxine hydrochloride
	N06A5	Milnacipran hydrochloride
Gabapentinoid	N02B0	Mirogabarine Besylate
	N03A0	Pregabalin
Antiepileptic	N03A0	Acetylphenetride
	N03A0	Ethosuximide

	N03A0	Carbamazepine
	N03A0	Gabapentin
	N03A0	Gamma-Amino Butyric Acid
	N03A0	Clonazepam
	N03A0	Clobazam
	N03A0	Stilipentol
	N03A0	Sulciam
	N03A0	Zonisamide
	N03A0	Topiramate
	N03A0	Trimetadione
	N03A0	Sodium valproate
	N03A0	Vigabatrin
	N03A0	Phenytoin
	N03A0	Phenytoin sodium
	N03A0	Phenytoin-Phenobarbital
	N03A0	Phenytoin-Phenobarbital Combination
	N03A0	Phenobarbital sodium
	N03A0	Primidone
	N03A0	Pregabalin
	N03A0	Perampanel hydrate
	N03A0	Fosphenytoin sodium hydrate
	N03A0	Midazolam
	N03A0	Lacosamide
	N03A0	Lamotrigine
	N03A0	Rufinamide
	N03A0	Levetiracetam
	N03A0	Lorazepam
	N03A0	Diazepam
	N03A0	Magnesium sulphate hydrate and dextrose
Antiarrhythmic	C01B0	Aplindin hydrochloride
	C01B0	Mexiletine hydrochloride
	C01B0	Lidocaine
	C01B0	Lidocaine hydrochloride
NMDA receptor antagonist	C04A1	Ifenprodil tartrate
	N01A2	Ketamine hydrochloride

	N04A0	Amantadine hydrochloride
	N07D9	Memantine hydrochloride
	R05D1	Dextromethorphan hydrobromide hydrate
Centrally acting muscle relaxant	M03B0	Afroqualone
	M03B0	Eperisone hydrochloride
	M03B0	Chlorfenecin carbamate
	M03B0	Tizanidine hydrochloride
	M03B0	Tolperisone hydrochloride
	M03B0	Baclofen
	M03B0	Pridinol mesylate
	M03B0	Methocarbamol

Supplement 5. Details of the diagnosis related to back pain

Diagnosis	Control<br>n=5,964	Case I<br>n=75	Case II<br>n=441
Acute lower back pain	190(3.2%)	1(1.3%)	8(1.8%)
Pain of thoracic spine	6(0.1%)	0(0.0%)	2(0.5%)
Chest/back pain	108(1.8%)	1(1.3%)	6(1.4%)
Musculofascial lower back pain	43(0.7%)	0(0.0%)	5(1.1%)
Cervicogenic headache	2(0.0%)	0(0.0%)	0(0.0%)
Cervical back pain	4(0.1%)	0(0.0%)	0(0.0%)
Cervical radiculopathy	8(0.1%)	0(0.0%)	2(0.5%)
Neck pain	280(4.7%)	5(6.7%)	9(2.0%)
Cervicobrachial neuralgia	3(0.1%)	0(0.0%)	0(0.0%)
Nuchal pain	3(0.1%)	0(0.0%)	0(0.0%)
Lumbosciatic neuralgia syndrome	7(0.1%)	0(0.0%)	1(0.2%)
Lower back pain	5,440(91.2%)	70(93.3%)	409(92.7%)
Lumbogluteal pain	11(0.2%)	0(0.0%)	1(0.2%)
Lumbar radiculitis	8(0.1%)	0(0.0%)	2(0.5%)
Lumboabdominal pain	8(0.1%)	0(0.0%)	1(0.2%)
Lower back pain of radicular origin	6(0.1%)	1(1.3%)	1(0.2%)
Sciatic neuralgia	96(1.6%)	0(0.0%)	14(3.2%)
Radiculitis	5(0.1%)	0(0.0%)	1(0.2%)
Spinal radiculopathy	2(0.0%)	0(0.0%)	0(0.0%)
Spine pain	1(0.0%)	0(0.0%)	0(0.0%)
Hip pain	79(1.3%)	2(2.7%)	12(2.7%)
Back pain	256(4.3%)	5(6.7%)	25(5.7%)
Chronic lower back pain	21(0.4%)	1(1.3%)	3(0.7%)
